# Influence of problem-solving ability and personality variables on the improvement and creativity of tactical decisions in basketball

**DOI:** 10.3389/fpsyg.2024.1450084

**Published:** 2024-09-30

**Authors:** Carlos Díaz-Rodríguez, Eugenio Pérez-Córdoba

**Affiliations:** ^1^Department of Physical Education and Sport, Faculty of Health and Sport, University EUNEIZ, Vitoria Gasteiz, Spain; ^2^Department of Experimental Psychology, Faculty of Psychology, University of Seville, Seville, Spain

**Keywords:** basketball, creativity, TACTICS, perception, decision making, personality, general intelligence

## Abstract

**Background:**

Basketball players are increasingly required not only to read the tactical actions that take place in a game but also to make correct tactical decisions. This includes greater creativity in the type of solutions they must provide when faced with various tactical situations. To acquire these skills, young players need training in which personality and general intelligence variables significantly influence the learning process.

**Methods:**

A Perceptual Tactical Training (PTT) program was implemented, correlated with the Eysenck Personality Questionnaire and the Raven’s Progressive Matrices test, to observe improvements in tactical perception and decision-making among young basketball players.

**Results:**

The PTT produced significant improvements in tactical solutions and tactical perception for all players. Players who scored high in general intelligence found it easier to perceive tactical situations but struggled to generate original tactical solutions. Conversely, players with high psychoticism and high general intelligence were more capable of creating innovative tactical solutions but did not excel in perceiving tactical game situations.

**Conclusion:**

Therefore, it is crucial to be aware of the individual differences in intelligence and personality traits among young players to enhance their tactical decision-making and creative abilities.

## 1 Introduction

The issue of creativity in sport is becoming increasingly important, considering that tactical systems are more complex than ever before, information media are multiplying, and the mobility of athletes is increasing exponentially. This leads to a heterogeneity of sporting scenarios and game combinations due to the diverse geographical origins of the athletes on the same team [Milennium Derecho Internacional Privado (DIPR), 2021]. Although there are insufficient studies in this field, many underline the importance of developing operative thinking, usually called tactical thinking in the sports sphere, which is the premise of creative performance (Aydin, 2019a; Aydin, 2019b; Bowers et al., 2014; Santos et al., 2017). There is evidence that creativity strongly influences work and sport practice (Klatt et al., 2019; Memmert et al., 2010; Conesa-Ros and Angosto, 2017; Marín et al., 2012), although more research is needed in the latter field.

Araújo (2006) follows, to some extent, the research line of Mahlo (1975) regarding creativity, though he more frequently refers to the term operative or tactical thinking. Several works have been published on creativity in sport (Martínez-Vidal and Díaz-Pereira, 2008; da Costa et al., 2018; Runco and Pritzker, 2011; Richard et al., 2017; Richard and Runco, 2020), but few have shown the explicit interest of the present research in addressing the topic in both a social and formative manner simultaneously.

One of the most controversial and difficult issues to address in the field of tactical thinking and creativity in sports is the decision-making process required to handle complex tactical situations within limited time frames (Furley and Memmert, 2015). It is crucial to remember that responses in sports need to be the most appropriate, despite the speed with which they must be executed (Hüttermann et al., 2019; Karaca and Aral, 2017). According to Memmert (2015), the most interesting aspect is the conflict athletes often face in sports games: they seek comprehensive, representative, and dynamic responses (in tune with the rapid pace of actions during a match) without sufficient time to consider the best course of action. Acting too quickly risks efficiency, while aiming for the most efficient solution might render the response obsolete in an instant, making it ineffective.

Memmert (2015) highlight that incorporating variables of intelligence or intellectual capacity is essential when designing psychological interventions to enhance the processes of perception and mental solutions in sports situations that demand high creativity (Alarcón, 2008; Alarcón et al., 2010; Alarcón et al., 2017).

Following the important theme of attention in developing effective tactical perceptions, Hüttermann et al. (2019) found an association between sport-specific divergent thinking in relation to tasks that included videos of real game situations, which were compared with performances of football players divided into two groups, with different levels of experience (amateurs and professionals).

The study of intelligence aims to measure the general factor by providing direct information on the individual magnitude of certain cognitive functions (observation and reasoning) and an evaluation of nearly all of them, representing the general intellectual capacity or common denominator of the totality of intelligence operations (Raven et al., 1991). This is one of the reasons why this theory and its resulting procedure were chosen for the present study.

Eysenck’s (1992) personality structure is a hierarchical model with four descriptive levels. The first level (the lowest) consists of observable and somewhat circumstantial responses that do not characterize the subject. The second level consists of habitual responses that the subject exhibits in similar situations. The third level includes behaviors that are habitually exhibited and can distinguish the subject, and the fourth level consists of general factors.

What is of interest is how athletes (in this case, basketball players) behave in certain ways in situations that demand selective perceptions, complex mental solutions, and, in a general sense, creativity that is supported not only by cognitive skills but also by dispositions and affective peculiarities. People with high scores in this dimension are more vulnerable to disorders traditionally called neurotic. These subjects may be more vulnerable to stressful situations, in which they are more likely to develop feelings of guilt, anxiety, low self-esteem, shyness, sadness, and emotionality (Pérez-Córdoba et al., 2003; Cantón et al., 2022). It is evident that the psychologist’s control of neuroticism is of great importance in the sports context, which, by nature, subjects the athlete to constant ergogenic, competitive, and self-affirmative pressures. Athletes with high neuroticism may find it difficult to navigate such situations and impose a proactive, optimistic, and confident response. In general, people who engage in sports activities early on show fewer manifestations of neuroticism because they learn more effective adaptive resources. The opposite pole of this dimension describes the athlete as capable of emitting adaptive responses according to the demands.

Another dimension of Eysenck’s model is introversion vs. extroversion, which is probably more commonly used in the area of sports because of its close relationship with levels of arousal and inhibition that are constantly tested in sports activities. Donado-Mazarrón (2019) analyzes that subjects with a high score in extroversion seek intense stimuli, take risks, and enjoy noisy environments. Conversely, subjects with low scores avoid stimulation, are cautious, serious, and prefer a type of life considered boring by extroverts. Hence, in sports, coaches and sport managers need to make good use of this scientific information on the personalities of athletes. Basketball is a good example in which the importance of this dimension in sports can be illustrated (González, 2019a,b).

Psychoticism is considered a dimension of normal personality, where extreme scores predispose individuals to psychotic disorders, bipolar disorder or schizophrenia, antisocial behavior, and psychopathy (Eysenck, 1992). The traits that characterize this dimension include aggressiveness, coldness, creativity, and impulsivity, as well as more controversial traits such as egocentrism and low empathy. There are several organic correlates associated with this dimension, but here we focus only on those that underline intelligence and creativity, in addition to the recognized sensation seeking of subjects with high psychoticism.

There is a predominant opinion that a certain presence of the psychoticism dimension in athletes contributes to increased creativity and instrumental aggressiveness necessary for good performance (Del Toro et al., 2013; González, 2019a,b), although some researchers have expressed doubts about this view (Iglesias, 2017).

## 2 Materials and methods

### 2.1 Ethical approval

Written informed consent was obtained from all participants prior to initiating the research. This study was approved by the Ethics Committee of the University of Sevilla and conducted following the guidelines established in the Declaration of Helsinki.

### 2.2 Participant

The sample consisted of 24 male basketball players aged between 16 and 17 years old, classified as juniors. They were purposefully divided into two groups: an experimental group comprising 16 members who underwent Tactical Perceptual Training (TPT), and a control group consisting of 8 members who received conventional training only over the course of thirteen weeks of TPT. The selection criteria for belonging to one group or the other was agreed with the coaches, who took into account that the control group should include players of all positions such as point guard, forwards and centre and that they should have average technical and tactical knowledge. All participants underwent both pre-experimental and post-experimental series of drills, with the results compared between the groups. Post-experimental testing using the Eysenck Personality Questionnaire (EPQ) was conducted for the control group after the conclusion of the study.

The selection of participants was based on purposive sampling of matched cases to ensure comparability between the experimental and control groups. All participants were exclusively male.

### 2.3 Variables

The study considered several key variables that influence the tactical and perceptual performance of basketball players:

#### 2.3.1 Meaningful tactical perception (STP)

Meaningful Tactical Perception is defined as the ability to extract essential information from the tactical situation a player faces, abstracting from the superficial and unnecessary, and responding effectively in minimal time (Díaz Rodríguez, 2021).

#### 2.3.2 Perceptual tactical training

Perceptual Tactical Training is defined as controlled exposure to images representing everyday situations in basketball games, aimed at developing meaningful tactical perception in the sport (Díaz Rodríguez, 2021).

#### 2.3.3 Intellectual capacity: abstract thinking and reasoning by analogies

Regarding intellectual capacity, the approach by Raven et al. (1991) was utilized, which conceptualizes intellectual capacity to compare forms and reason by analogies, independent of acquired knowledge.

#### 2.3.4 Neuroticism

Neuroticism follows Eysenck’s (1992) perspective as a personality trait revealing low self-control, suggestibility, lack of sociability, and an inability for volitional effort. It is assessed on a graded scale from low neuroticism (absence of these characteristics) to high neuroticism, characteristic of individuals diagnosed with neuroticism.

#### 2.3.5 Extroversion

Extroversion is defined according to Eysenck’s (1992) framework as a personality trait revealing high sociability, communicativeness, and a favorable balance in arousal-inhibition processes. Extroverts tend to perform better in high-stress situations, while introverts excel in monotonous or low-demand situations.

#### 2.3.6 Psychoticism

Psychoticism is conceptualized based on Eysenck’s (1993) perspective as a personality trait revealing aggressiveness, coldness in the face of challenges, a tendency toward bizarre or unpredictable behavior, hostility, and divergent thinking. It is assessed on a graded scale from low (absence of these characteristics) to high, characteristic of individuals diagnosed with psychosis.

### 2.4 Instruments

#### 2.4.1 Instruments for tactical perceptual training (TPT)

The TPT consists of images depicting 10 basketball players engaged in specific game actions requiring technical-tactical decisions. These images form an experimental series of 25 slides presented sequentially, each displayed for a very short period of time (maximum 3 s). As the sessions progress, the presentation time is reduced by 0.2 s until reaching a minimum exposure time of 2 s (e.g., 3 s., 2.8 s., 2.6 s., 2.4 s., 2.2 s., and 2.0 s.). The purpose of this controlled time reduction is to condition players to develop meaningful tactical perception, focusing on essential tactical information while disregarding irrelevant details, thus enabling them to formulate precise and effective responses in the shortest possible time. The instrument is structured around two main phases: (a) perception and analysis of the situation; (b) tactical decision-making. Its aim is to facilitate the spontaneous identification of critical cues that prompt accurate responses to each presented image.

#### 2.4.2 Raven’s progressive matrices test

The Progressive Matrices test (Raven et al., 1991) is designed to assess the “G” factor, which represents general intelligence essential for problem-solving across various cognitive tasks. The test emphasizes analytical reasoning, perception, and abstraction abilities through analogical reasoning, independent of specific cultural knowledge or prior experience. An example of a recent adaptation can be found in Te Nijenhuis et al. (2019).

#### 2.4.3 Eysenck personality questionnaire (EPQ-R)

The EPQ-R is an administered test that evaluates three dimensions of personality:

- **Neuroticism (N):** Provides information on personal stability, anxiety levels, potential depression, and other related traits.
- **Extraversion (E):** Assesses sociability and arousal levels, ranging from lively and adventurous to quiet and withdrawn, with Introversion as its opposite.
- **Psychoticism (P):** Measures behavioral traits including aggressiveness, unconventionality, empathy, and propensity for conflict.
- **Self-portrait distortion scale (L):** Aims to assess response sincerity or comprehension level. Scores for each dimension are determined according to established test norms (Hernán-Gómez and de la Peña, 2018).

### 2.5 Procedure

The experimental design runs over thirteen consecutive weeks. The TPT was conducted three times per week (Monday, Wednesday, and Friday) in a classroom inside a school hall equipped with a projector. The test administration time was around 30 min. These days were designated because the players had training sessions with their team and could meet before the basketball training session. All individuals participated between September 2018 and May 2019, while the pre-design phase took place between September 2017 and August 2018.

Table 1 shows the partial planning of the work sessions, illustrating the distribution of sessions from the second to the fifth week, with the first week dedicated to the pre-experimental series. In week 1, the Raven’s Progressive Matrices Test, the 2.4.3 Eysenck Personality Questionnaire (EPQ-R) and TPT were administered. In week 13, the TPT was retested and the results were compared. It is observed that if there is a higher score in the TPT results, there has been an improvement in tactical learning and connect it with the data from the questionnaires carried out in week 1.

**Table 1:** Experimental planning design.

Week 1	Week 2	Week 3	Week 4	Week 5
Monday Implementation sequence Pre-experimental	Block 1 *3 seconds*	Training break TPT *(just basketball court training)*	Block 2 *2.8 seconds*	Training break TPT *(just basketball court training)*
	Monday Experimental sequence 1 Feedback Sequence 1		Monday Experimental sequence 4 Feedback Sequence 4	
	Wednesday Experimental sequence 2 Feedback sequence 2		Wednesday Experimental sequence 5 Feedback sequence 5	
	Friday Experimental sequence 3 Feedback sequence 3		Friday Experimental sequence 6 Feedback sequence 6	

Week 6	Week 7	Week 8	Week 9
Block 3 *2.6 seconds*	Training break TPT *(just basketball court training)*	Block 4 *2.4 seconds*	Training break TPT *(just basketball court training)*
Monday Experimental sequence 7 Feedback sequence 7		Monday Experimental sequence 10 Feedback sequence 10	
Wednesday Experimental sequence 8 Feedback sequence 8		Wednesday Experimental sequence 11 Feedback sequence 11	
Friday Experimental sequence 9 Feedback sequence 9		Friday Experimental sequence 12 Feedback sequence 12	

Week 10	Week 11	Week 12	Week 13
Block 5 *2.2 seconds*	Training break TPT *(just basketball court training)*	Block 6 *2.0 seconds*	Friday Implementation sequence Post-experimental
Monday Experimental sequence 13 Feedback sequence 13		Monday Experimental sequence 16 Feedback sequence 16	
Wednesday Experimental sequence 14 Feedback sequence 14		Wednesday Experimental sequence 17 Feedback sequence 17	
Friday Experimental sequence 15 Feedback sequence 15		Friday Experimental sequence 18 Feedback sequence 18	

The subsequent weeks maintain the same work pattern until week 12, during which all the experimental series are conducted along with their respective feedback sessions. In week 13, when the experiment concludes, a post-experimental series is carried out, following the same dynamics as the first week with the pre-experimental series.

### 2.6 Statistical analyses

The statistical procedures used were: Kolmogorov–Smirnov test to verify the normal distribution of the data; calculation of means, standard deviations and minimum-maximum intervals of correct perceptions for the two pilot groups; t-tests of differences between means, paired observations, to estimate the significance of the differences. By means of the simple regression analysis model, the predictive values of the personality qualities studied were studied.

#### 2.6.1 Verification of the TPT working hypothesis

The working hypothesis was verified in two parts, corresponding to the two questions that the players had to answer for each slide:

- Tactical Perception: What is happening on the court at the moment?
- Tactical Solution: What tactical solution would you give to this situation?

The question related to the tactical solution takes into account the playing philosophy of both the player’s own team and the opposing team.

For both parts, *t*-tests for differences between means (paired observations) were applied, as this test best suits the interest of confirming possible differences when dealing with an ordinal scale as used in this part of the research.

#### 2.6.2 Validation of tactical perception

According to the results of the experimental group, the tactical perceptions and mental solutions were evaluated in each of the 25 pre- and post-experimental slides, and the values were recorded in a database for processing using SPSS version 24.

Descriptive statistics were obtained for each of the indicators referred to in this methodological chapter. The values obtained for each response of perception and mental solution were very similar to those obtained in the experiment itself, as will be seen later in the results chapter.

The t-test for differences between means (paired observations) was used to determine the differences in the number of correct tactical perceptions between the experimental group (who received the TPT) and the control group (who did not receive it), adopting a *p*-value equal to or less than 0.05 to determine those that were significant.

Additionally, using Pearson’s rank correlation matrix, the number of correct tactical solutions was studied concerning the first question regarding the significant tactical perception, the PIR values, and those obtained in each of the performance rating scales, and the correlations were taken into account.

#### 2.6.3 Validation of the tactical solution

To develop this objective, the 16 subjects of the experimental group were previously evaluated to determine the level of abstract thinking or reasoning by analogies (Raven’s Progressive Matrices Test), and the degree of neuroticism, extroversion, and psychoticism using Eysenck’s EPQ Test.

Subsequently, all evaluations regarding the tactical perceptions on each experimental slide (evaluated as correct or incorrect) and the solutions issued for each of them (evaluated as incorrect, incomplete, correct, or original positive) were collected.

Using the simple regression analysis model, the predictive values of the personality qualities studied with respect to each indicator of perception and mental solution for each slide were examined, adopting a *p*-value equal to or less than 0.05 to determine those that were significant.

## 3 Results

### 3.1 Results of abstract reasoning ability as a predictor of mental solutions

By means of a regression analysis (Table 2), it was observed that high scores in the Progressive Matrices Test in the experimental group that received the TPT were able to predict a greater number of successes in the tactical perception promoted by the slides.

**Table 2:** Regression analysis to determine the predictive value of progressive matrix test scores at various increments after receiving ETP.

Variable	R	df	F	*p*
Delta (increase) of accurate perceptions	0.581	15	7.134	0.018*
Delta (increase) of successes of successful mental solutions	0.358	15	2.052	0.174 (n.s.)
Delta (increase) of original mental solutions	0.494	15	4.508	0.05

*Indicates that the data confirm the improvement observed in the study.

The correct answers to the second research question, “What tactical solution would you give to this situation?” were not significantly associated this time with intellectual capacity *per se*, which reveals that without knowledge and active experience, intelligence does not advance much further. As shown in the following table, a regression analysis with the variable intellectual ability as a predictor did not reach statistical significance against the number of correct solutions as the dependent variable.

In other words, the ability to find relations through analogies and to develop abstract reasoning favors the identification of what is happening on the field but not the solution that is issued.

In the Table 2 regarding the result of a regression analysis to determine the predictive value of the Progressive Matrices Test on the increase of correctness in the phase of perception and analysis of the situations contained in the TPT slides, the aforementioned calculation revealed that the ability to develop abstract reasoning and thinking by analogies in the sample studied was able to predict significantly (*p* = 0.018) a better use of the TPT for the tactical perception phase.

As shown in Table 2, the better intellectual performance showed an interesting relationship with the ability to find originally positive solutions to the challenges imposed by the experimental series. The aforementioned relationship was significant (*p* = 0.05), which allows us to understand that intellectual capacity plays a role in identifying what is happening in the game scenario based on elements that deviate from the canons and expectations, incorporating elements of high originality into cognitive management.

### 3.2 Results for psychoticism and other personality variables as predictors of tactical or mental solutions

As can be seen in the following Table 3, the personality variables that showed significant correlations with the Tactical Perceptual Training (TPT) results were abstract thinking and psychoticism, while neuroticism and extroversion were not significant.

**Table 3:** Matrix of correlations between personality variables (intellectual ability, neuroticism, extroversion and psychoticism) and correct and original solutions at the end of the thirteen weeks of ETP.

Variable	Raven	Neurot.	Extrov.	Psicot.	Sol. corr. post	O+Post
Raven	–	0.712	0.336	0.099	0.419	0.000
Neurot.	0.712	–	0.062	0.176	0.223	0.111
Extrov.	0.336	0.062	–	0.690	0.548	0.841
Psicot.	0.099	0.176	0.690	–	0.036*	0.122
Sol. Corr. Post	0.419	0.223	0.548	0.036*	–	0.100
O+Post	0.006**	0.111	0.841	0.122	0.100	–

*p* less than 0.05. *Indicates that Psychoticism showed a significant relationship with the number of correct solutions in the post-experimental evaluation. **Indicates that the score on the Progressive Matrices Test (abstract thinking or reasoning by analogies) was very significantly related to the number of original positive solutions provided in response to the slides after the experimental situation.

The following aspects were tested:

- The Progressive Matrices Test score (abstract thinking or reasoning by analogies) was very significantly related to the number of positive originals emitted in front of the slides after the experimental situation.
- Psychoticism showed a significant relationship with the number of correct solutions in the post-experimental evaluation.

#### 3.2.1 Personality variables and tactical performance

Personality variables also had a certain impact on tactical performance, as illustrated in the Table 3. It shows the correlations between the observable components of the final version of the performance scales for the experimental group and the two personality qualities that have shown the greatest influence in this study.

In addition to the greater benefits of TPT in the experimental group referred to above when analyzing the PIR values, these results will deepen the directions of improvement and are reflected in the correlations below. Considering that these scales were evaluated by experts using ordinal statistics, a study of correlations made it possible to specify the particular benefits of TPT on these scales.

#### 3.2.2 Neuroticism, extroversion, and performance rating scales in the experimental group

The variables neuroticism and extroversion did not show the same significance in the present study as intelligence and psychoticism (Table 3). This does not mean that they lack influence on the behavior of the athletes; it only seems to indicate that when testing the cognitive capacity necessary to discern what happens in an interactive, synergistic, and complex tactical context such as in basketball games (and to do so in conditions of more intense time deficit than in real practice, as in the case of those arranged in the last three weeks of the TPT: times of 2.4, 2.2, and 2.0 s), these variables do not play the role of intelligence and psychoticism.

## 4 Discussion

The primary objective of this study was to examine how problem-solving ability and personality variables influence the improvement and creativity of tactical decisions in youth basketball players.

The results indicated that the Perceptual Tactical Training (PTT) program led to significant improvements in both tactical solutions and tactical perception for all players. Additionally, players with higher abstract reasoning ability demonstrated greater efficiency in perceiving tactical situations, while those with high levels of psychoticism and general intelligence were more capable of generating innovative tactical solutions.

Regarding abstract thinking, its relevance for the development of technical and tactical skills is acknowledged (Aydin, 2019a). Although it is not a sufficient condition for developing the levels of creativity needed in specific activities such as sports and, above all, for achieving significant tactical perceptions of high quality and speed, it is clear that having medium to high intellectual capacity facilitates the use of Tactical Perceptual Training (TPT). However, without theoretical-tactical knowledge and active sporting experience, an intelligent athlete would not be able to access the tactical concept presented with the relevance of an expert, as is needed in competitions. Creative thinking is nourished by this basic intelligence, which becomes a premise for reaching higher levels of tactical thinking (Araújo, 2006).

Intelligence endowed with the aforementioned knowledge and skills may not be enough to achieve the expression of talent that ensures success; other personality characteristics are needed. Psychoticism appears in the present study to be significantly associated with the number of successful tactical perceptions under time constraints during the game days (Araújo, 2006).

Several of the seven factors that make up the classic psychoticism dimension of Eysenck and Eysenck (2008) stand out as potential contributors to the creativity stimulated by the designed TPT: impulsivity, antagonism, and non-conformism. Others related to the low socialization of subjects with high psychoticism were not taken into account here, as they do not fit the essentially cognitive approach to the research. However, the three mentioned are sufficient to support the creative projection that allows the use of the perceptual tactical training that the basketball players examined here received.

According to Pelechano (1997), people with high psychoticism can be original in this process of coordinated activation of cognitive resources, which memory stores in a relatively passive way. The contribution of psychoticism includes the so-called non-inclusive style of thinking (Pelechano, 1997) and a larger arsenal of ideas on which to build a conclusion, which allows for the emergence of more innovative, rare, and creative responses than those with a more conventional approach to the problem. This may be part of the basis of creativity, associated with the psychotic dimension.

The relationship between psychoticism and so-called creative achievement in artists has been tested. Götz and Götz (1979) found that artists had significantly higher psychoticism scores than non-artists, contributing to Eysenck’s (1993) confirmation that real artistic achievement is associated with psychoticism. It is not difficult, then, to accept the scientific evidence that something similar occurs with high-achieving athletes. Top and Akil (2018) found that males who scored highest in scholastic, scientific/mechanical, and artistic creativity also scored highest in psychoticism.

However, research does not always support the role of psychoticism in sports activity; some studies report no significant differences between this variable and performance (Bernacka et al., 2016). One possible explanation is that those studies lacked the experimental character of the present one and were limited to descriptive/comparative studies of men and women, practitioners and non-practitioners, without delving into the essence of self-regulatory elements such as those that come into play when an athlete must interpret the content of a tactical slide, develop complex and rapid cognitive processes to detect relevant signals, and disregard accessory ones in the shortest time. In the post-experimental games, it was shown by a group of experts that these skills were also manifested in the specific sport activity.

Bernacka et al. (2016) have reported that the level of psychoticism was lower in weightlifters and ju-jitsu practitioners, although they acknowledge that in other studies conducted with women in combat sports, non-conformism, a trait closely related to psychoticism, appears with greater presence (Burdizicka and Goral, 2014, cited by Bernacka et al., 2016).

Another reason that may explain the scarce scientific attributions to psychoticism as a personality variable capable of generating creativity in athletes is that many authors do not study the dimension itself but rather indicators related to this dimension since the first and efficient studies conducted by Eysenck and other authors mentioned above. For example, greater instrumental aggressiveness and “toughness” have been observed in creative athletes, qualities clearly associated with psychoticism. Memmert (2011, 2015) has done relevant and sustained work on the possible ways in which creativity can contribute to the world of sport, defining it as “unusual, innovative, statistically rare, or even unique behavior in solutions to a sport-related situation” (Memmert, 2011, p. 373), which is an obvious way to relate it to the psychoticism found in our work.

In contrast to convergent thinking, which generally leads to conventional and correct solutions, divergent thinking, characteristic of high psychoticism, is defined as the process of generating many alternative ideas (Williams et al., 2016) and occurs in a universe that does not initially recognize boundaries or exclusions. This notion has become key to the mechanism underlying creativity; it exemplifies how the creative brain acts cognitively and is a variable that can be quantified. Divergence means considering different perspectives, looking for more than one answer, dismantling rigid schemes, and not relying on single, prior assumptions, “that is, trying out, making new associations, selecting in unusual ways, restructuring the apparently unusual or useless, going down unexpected paths, and testing to produce something new or unknown” (Torrance and Myers, 1976, p. 11).

It is clear that the pragmatism and instrumental aggressiveness found in subjects with higher psychoticism endow these basketball players with a better “sixth sense,” a term used in sports to refer to the early discovery of tactical signals after the training received. This explains why TPT has a significant impact on athletes with higher psychoticism, similar to its impact on intelligence. As García Peñas et al. (2021) state, sport constitutes “a very favorable context for innovation, as it usually entails a persevering task of improving the skills that come into play.” Other authors emphasize the innovative attitude that a high-performance athlete must have (Hackfort and Schinke, 2020), and at the core of such an attitude is the creativity of the subject with a propensity for psychoticism. Memmert (2015) defines it as “unusual, innovative, statistically rare, or even unique behavior in solutions to a sport-related situation”.

This unusual and innovative attitude makes the basketball player with high psychoticism a greater beneficiary of TPT, even though it also benefits others. As Williams et al. (2016) assert, the convergent thinking of low-psychoticism subjects leads to conventional and “correct” solutions, while the divergent thinking of high-psychoticism basketball players generates many “alternative ideas,” allowing them to take better advantage of the TPT designed for this research.

This finding can become a resource to develop creativity, which is crucial for good tactical performance. It can find through TPT a way to stimulate the brain from a cognitive point of view, allowing for a quantitative approach complemented with qualitative assessments observed by experts during real basketball games. The present research can enrich the understanding of the relationships between intelligence and creativity to assimilate perceptual tactical training, increase performance, and facilitate criteria for selecting athletes with certain personality characteristics to enhance creativity in ball game sports.

Sport psychology needs to explore and scientifically substantiate observations such as these, and it is not possible to do so solely with descriptive studies, translations, psychometric adaptations, and mass applications, but with correct and accurate explorations of variables, as close as possible to the specific sport activity.

## 5 Limitations and future research

The limitations encountered and future research that could be undertaken can be summarised as follows:

1. **Sample size limitation:** The size of the sample restricts the generalizability of findings to basketball players of different ages and backgrounds.
2. **Inclusion of psychophysiological evaluations:** Future studies could incorporate psychophysiological evaluations such as heart rate monitoring, skin conductance, thermometry, and other relevant measures. These could provide insights into physiological responses during perceptual tactical training sessions.
3. **Utilization of advanced information technologies:** Leveraging advancements in information technology, future research could explore the use of moving images. This approach could enhance the complexity of tactical scenarios presented, thereby enriching the perceptual training experience.

These considerations aim to address current limitations and propose avenues for further enhancing the effectiveness and scope of perceptual tactical training in basketball.

## 6 Conclusion

Abstract reasoning ability and a predisposition toward psychoticism play a key role in enhancing tactical perception through perceptual tactical training (PTT).

Players with higher abstract reasoning ability demonstrated greater accuracy in perceiving tactical situations, highlighting the importance of analogical reasoning in identifying game dynamics. However, this ability alone does not guarantee the generation of optimal tactical solutions, emphasizing the need to complement intelligence with practical knowledge and experience.

Although the PTT improved participants’ perception abilities, it was not sufficient to directly enhance the accuracy of tactical solutions. This suggests that tactical decision-making requires a multifaceted approach that combines intelligence, experience, and deliberate practice.

The results also suggest that psychoticism, combined with abstract reasoning, facilitates the rapid identification of critical aspects in tactical situations, which can be beneficial for decision-making in basketball.

## Data Availability

The raw data supporting the conclusions of this article will be made available by the authors, without undue reservation.
